# Mesenchymal Stromal Cells “Think” Globally, but Act Locally

**DOI:** 10.3390/cells12030509

**Published:** 2023-02-03

**Authors:** Günther Eissner

**Affiliations:** Systems Biology Ireland, School of Medicine, Conway Institute, University College Dublin, D04 V1W8 Dublin, Ireland; guenther.eissner@ucd.ie

In this Special Issue of *Cells*, entitled “Immunomodulation by Mesenchymal Stem Cells 2020”, you can find five excellent papers on the role of mesenchymal stem/stromal cells (MSCs) in immunomodulation, which also includes regenerative processes, such as wound healing.

MSCs are fibroblast-like multipotent progenitor cells with immunosuppressive properties in vitro and in vivo [1]. MSCs are increasingly being used in clinical trials, e.g., for chronic graft-versus-host disease following haematopoietic stem cell transplantation [2]. Although the precise mechanism of action (MOA) of these cells in various clinical applications is not fully understood, recent progress has been made in the functional characterisation of MSCs and their extracellular vesicles, especially in the perinatal field [3,4,5,6], which can easily been translated to adult MSCs.

MSCs cannot only suppress immune responses, but also induce them, e.g., humoral immunity. In this Special Issue, Bikorimana et al. describe that an immunoproteasome complex expressing MSCs could produce antibodies in response to ovalbumin stimulation, which has an antitumour function in vivo [7]. Of note, humoral immunity by MSCs requires the presence of phagocytes providing efferocytosis [7]. Another focus of this Special Issue lies in adipose tissue-derived MSCs (ASCs). A very good example for a local action of MSCs is given by Kuca-Warnawin et al., who point out that ASCs from ankylosing spondylitis patients could—when co-cultured with peripheral blood mononuclear cells—enhance the generation of anti-inflammatory regulatory T cells (Treg) and could downregulate interferon γ expression [8]. Two more papers on ASCs from Karen Bieback’s group also address Treg generation and its MOA. Fiori et al. report that ASCs generally inhibit CD4+ T cell proliferation through the local action of indoleamine 2,3-dioxygenase (IDO), but could stimulate the differentiation between CD127+ and FoxP3+ Treg at the same time. Importantly, ASCs only had these effects when co-cultured with unstimulated PBMC [9]. In an important comparison of MSCs from different sources, Torres Crigna et al. found ASCs to be superior in their T-cell antiproliferative effect to MSCs from bone marrow or cord blood [10]. Interestingly, only conditioned medium, but not EV from ASCs, could exert similar effects. Again, IDO was identified to be responsible for this type of immunomodulation. Furthermore, human ASCs could not inhibit murine lymphocyte proliferation, bringing into question interspecies experimental set-ups for the functional testing of MSCs in general [10]. The final addition to this Special Issue of *Cells* is a comprehensive review by Raquel Guillamat-Prats about the role of MSCs in wound healing, with a focus on diabetes and fibrosis. Of note, MSCs and their derivatives have different functions in different steps of the wound repair process [11].

In this Special Issue of *Cells*, we have learned about the critical role of IDO in the mechanism of MSC action and how important the fine-tuning of local MSC actions is for an effective therapeutic approach. Further work is needed to elaborate how MSC derivatives can be used in the clinic.
